# Development and implementation of “Check of Medication Appropriateness” (CMA): advanced pharmacotherapy-related clinical rules to support medication surveillance

**DOI:** 10.1186/s12911-019-0748-5

**Published:** 2019-02-11

**Authors:** Charlotte Quintens, Thomas De Rijdt, Tine Van Nieuwenhuyse, Steven Simoens, Willy E. Peetermans, Bart Van den Bosch, Minne Casteels, Isabel Spriet

**Affiliations:** 1 Department of Pharmaceutical and Pharmacological Sciences, KU Leuven, Herestraat 49, B-3000 Leuven, Belgium; 2 Pharmacy Department, University Hospitals Leuven, Herestraat 49, B-3000 Leuven, Belgium; 3 Department of Microbiology and Immunology, KU Leuven, Herestraat 49, B-3000 Leuven, Belgium; 4 Department of General Internal Medicine, University Hospitals Leuven, Herestraat 49, B-3000 Leuven, Belgium; 5 Department of Public Health and Primary Care, KU Leuven, Herestraat 49, B-3000 Leuven, Belgium; 6 Department of Information Technology, University Hospitals Leuven, Herestraat 49, B-3000 Leuven, Belgium

**Keywords:** Check of medication appropriateness (CMA), Clinical validation of prescriptions, Clinical decision support (CDSS), Computerized physician order entry (CPOE), Medication surveillance, Clinical rules

## Abstract

**Background:**

To improve medication surveillance and provide pharmacotherapeutic support in University Hospitals Leuven, a back-office clinical service, called “Check of Medication Appropriateness” (CMA), was developed, consisting of clinical rule based screening for medication inappropriateness. The aim of this study is twofold: 1) describing the development of CMA and 2) evaluating the preliminary results, more specifically the number of clinical rule alerts, number of actions on the alerts and acceptance rate by physicians.

**Methods:**

CMA focuses on patients at risk for potentially inappropriate medication and involves the daily checking by a pharmacist of high-risk prescriptions generated by advanced clinical rules integrating patient specific characteristics with details on medication. Pharmacists’ actions are performed by adding an electronic note in the patients’ medical record or by contacting the physician by phone. A retrospective observational study was performed to evaluate the primary outcomes during an 18-month study period.

**Results:**

39,481 clinical rule alerts were checked by pharmacists for which 2568 (7%) electronic notes were sent and 637 (1.6%) phone calls were performed. 37,782 (96%) alerts were checked within four pharmacotherapeutic categories: drug use in renal insufficiency (25%), QTc interval prolonging drugs (11%), drugs with a restricted indication or dosing (14%) and overruled very severe drug-drug interactions (50%). The emergency department was a frequently involved ward and anticoagulants are the drug class for which actions are most frequently carried out. From the 458 actions performed for the four abovementioned categories, 69% were accepted by physicians.

**Conclusions:**

These results demonstrate the added value of CMA to support medication surveillance in synergy with already integrated basic clinical decision support and bedside clinical pharmacy. Otherwise, the study also highlighted a number of limitations, allowing improvement of the service.

**Electronic supplementary material:**

The online version of this article (10.1186/s12911-019-0748-5) contains supplementary material, which is available to authorized users.

## Background

Inappropriate prescribing has been shown to be an independent risk factor for adverse drug events (ADE) and several studies reported that ADEs can cause hospitalization [1]. Moreover, half of these hospital admissions are preventable [2, 3]. Different strategies were already described to prevent inappropriate prescribing, i.e. 1) a clinical pharmacist attending physician rounds, 2) computerized physician order entry (CPOE) and 3) CPOE with clinical decision support system (CDSS) [4].

In order to augment the safety and quality of patients’ therapy, front-office bedside clinical pharmacy services were set up in many European countries, after these were implemented in the UK, Canada and the USA since the ‘70s [5]. Bedside, embedded clinical pharmacists are typically involved in medication reconciliation and review, medication counselling at discharge and targeted projects improving medication use [6]. However, due to limited healthcare budget, which is the case in many European countries, bedside clinical pharmacy services are not implemented on a hospital-wide basis but restricted to high-risk patient populations such as geriatric patients characterized by polypharmacy, critically ill patients or patients admitted at the emergency department or on surgical wards with rapid patient turnover [5, 6].

With the increase in information and implementation of CPOE, medical and treatment data are available in a structured way which led rapidly to embedding basic and advanced CDSS in the CPOE (CPOE/CDSS) in order to support appropriate prescribing [7–9]. During the prescription process, basic CDSS analyse the data in the CPOE based on clinical rules and provide alerts automatically signalling clinical problems such as drug-drug interactions (DDI), basic dosing guidance, drug-allergy checking, duplicate therapy, etc. Nevertheless, basic CDSS is only providing support at the step of prescribing and not during follow-up treatment, without taking into account relevant biochemical parameters [9]. More advanced CDSS also contain clinical rules combining several sources of information on the characteristics of individual patients such as laboratory values [9–13].

Studies have shown a clear benefit of CPOE/CDSS with a significant decrease in prescription errors and ADEs [7, 14–18]. Additionally, Rommers et al. showed the added value of advanced clinical rules in preventing ADEs when used in combination with basic CDSS [10]. Conversely, the rise in health information technology has induced new pitfalls such as ‘alert fatigue’, i.e. the ignorance of both relevant and non-relevant alerts by health professionals because of moderate to low clinical relevance of some alerts, especially for basic clinical rules [18–20]. Eppenga et al. demonstrated the improved (but still not optimal) clinical relevance of medication alerts when including more patient-related characteristics [13]. Furthermore, sophisticated CDSS requires well-established digital communication, an effective integration of data and frequent updates [19].

In the University Hospitals Leuven, Belgium, medication surveillance is supported by both bedside clinical pharmacists on high-risk wards (geriatrics, pediatrics, emergency department, trauma surgery, abdominal surgery and septic orthopedic surgery) and a basic CPOE/CDSS. Given the limitations of the basic CDSS (i.e. alert fatigue; no integration of biochemical parameters) and the limited implementation of bedside clinical pharmacy services along with the aim for medication surveillance and review on a hospital-wide basis, which is driven by the hospital accreditation standards, University Hospitals Leuven has developed a new back-office clinical service: the “Check of Medication Appropriateness” (CMA).

This service consists of advanced clinical rule alerts aiming exclusively at hospital pharmacists, followed by pharmacists’ performed actions aiming at physicians in case of medication inappropriateness. The objective of this manuscript is to describe the development of CMA and to evaluate the preliminary results of this new clinical service.

## Methods

### Setting

The University Hospitals Leuven is a 1995-bed academic tertiary care centre. The Hospital’s Information System (HIS) with patients’ electronic medical records for non-critically ill patients integrates patient-specific data (including demographics, laboratory values, clinical parameters), surgical and radiology reports, physicians’ and nursing notes, CPOE and medication orders. The CPOE system is integrated with a CDSS, which provides support at the moment of prescribing by checking for DDIs, drug-food interactions, drug use during pregnancy and lactation, allergies, maximum dosage and therapeutic duplication. The safety alerts for DDIs are based on a national database (DelphiCare®, APB, Belgium). DDIs are categorized in three groups: very severe DDIs, severe DDIs and other DDIs (with minor clinical relevance). The prescribing physician only has to overrule the alerts for very severe DDIs, and if so, should provide a motivation. Prescribing for patients admitted on the intensive care unit (ICU) is carried out in another software system without integrated CDSS.

### Set-up of CMA service

The objective of CMA is to validate the treatment of patients at risk for potentially inappropriate medication (PIM), including drug-related problems (DRPs) and ADEs by combining structured data available from HIS and by using standardized algorithms, also referred to as clinical rules. The development process (realized with 0.2 full-time equivalents (FTE) pharmacist) consists of three phases: (1) development of the clinical rules, (2) development of the CMA, and (3) validation of the service.

#### Development of the clinical rules

The need for specific clinical rules was determined by a multi-method approach. Patients at risk for PIMs were identified based on bedside clinical pharmacy experience and patient safety incident reports. The clinical rules were defined based on literature and (inter)national guidelines (e.g. IDSA guideline for the management of catheter-related infections [21], EHRA guideline on the use of new oral anticoagulants [22], etc.). All clinical rules were reviewed in a multidisciplinary team consisting of clinical pharmacists and medical staff, and approved by the hospital board and Pharmacy & Therapeutics committee (P&T) to finally result in a definite set of 78 advanced clinical rules which were considered to be relevant for our hospital practice (Additional file 1). These clinical rules were grouped in five pharmacotherapeutic categories i.e. 1) overrules of alerts for very severe DDIs generated by the CDSS, 2) drugs with a restricted indication or dosing, 3) medication potentially leading to biochemical changes, 4) potential sequential therapy (intravenous (IV) to oral switch), and 5) others. The third category, i.e. medication use potentially leading to biochemical changes, is further divided in six subcategories as shown in Table 1.

**Table 1:** Pharmacotherapeutic categories and subcategories used to define the clinical rules^a^

	Category (and subcategories)	Example of a clinical rule
1	Overrules of alerts for very severe DDIs generated by the CDSS	Reduced effect of valproic acid by carbapenems leading to an increased risk of convulsions
2	Drugs with a restricted indication or dosing	Patient with high dose meropenem
3	Medication use potentially leading to biochemical changes	
	* Drug use in renal insufficiency	Patient with a CrCl < 30 ml/min and treated with metformin
	* Drugs with high potential of QTc interval prolongation	Patient with a QTc > 450/470 ms and treated with haloperidol
	* Drug use associated with hyperpotassemia	Patient with a K > 5.5 mmol/L and treated with an ACE inhibitor
	* Drug use associated with hypopotassemia	Patient with a K < 3.5 mmol/L and treated with flucloxacillin without potassium supplementation
	* Drug use associated with supratherapeutic INR	Patient with a supratherapeutic INR (INR > 4) and treated with a VKA
	* Drug use associated with bone marrow suppression	Patient with an absolute neutrophil count < 1.5*10^9^/L and treated with clozapine
4	Potential sequential therapy for bio-equivalent drugs	Potential sequential therapy for levofloxacine
5	Others	Patient treated with non-crushable drugs administered through enteral feeding tube

^a^*DDI* drug-drug interaction, *CDSS* clinical decision support system, *INR* international normalized ratio, *CrCl* creatinine clearance, *ACE* angiotensin converting enzyme, *VKA* vitamin K antagonist

#### Development of the CMA

The algorithms are formulated as ‘if-then’ rules and the CMA system, based on a Microsoft Access database, generates alerts invoked by specific triggers (‘clinical rule alert criteria’) based on screening the available data in the electronic medical record and CPOE. In this way, medical records along with treatment schemes are screened, both in non-critically ill hospitalized patients and patients admitted at the day care hospital. Screening and generation of the alerts take place daily at 12 am.

For the category ‘potential sequential therapy’, the algorithm is automatically selecting patients with presumed intact gastrointestinal absorption and a prescription for an IV bio-equivalent drug. An electronic note explaining the potential IV to oral switch is automatically provided in the patient’s medical file. For the other four categories, the results of the screening, the clinical rule alerts, are listed on a structured worklist. This worklist is assessed on a daily basis (0.5 FTE) by a trained hospital pharmacist for appropriateness of treatment. For each clinical rule, a user-friendly standardized flowchart or decision tree was drawn, on which the hospital pharmacist can rely while validating the prescriptions. When deemed necessary, an action on the alert is carried out by leaving an electronic note for the treating physician in the patient’s medical record. The message of the note is predefined for each clinical rule in the concerning flowchart. In case of a potentially severe ADE, the physician is also contacted by phone next to the electronic note.

#### Validation of the CMA

To ensure that the system selects the patients at risk for predefined PIMs, a validation was performed by two different methods. First, fictive patients were used to create fictive medication orders in the CPOE to test if the medication orders are screened appropriately based on the predefined algorithms. Through the use of a set of test patients, the sensitivity of the system can be evaluated. Second, the CMA was performed behind-the-scene on hospitalized patients. The medical record of the patients who were listed on the worklist was checked manually to search for false positive results to evaluate the specificity of the system. Based on this validation, the structure of the clinical rules was adapted and optimized to reduce the number of false positive and false negative results.

Twenty-one hospital pharmacists are participating in the CMA service. To assess if the validation is carried out in the same way, interrater reliability using kappa statistics was determined. The kappa (ƙ) value, a chance-corrected index of agreement, can range from − 1 (complete disagreement) to + 1 (perfect agreement). The trained hospital pharmacists had to check ten clinical rule alerts, using the available flowcharts. An average ƙ of 0.79 was obtained, which corresponds to an adequate agreement in performing the checking of PIMs based on the standardized flowcharts.

### Study design

The impact of CMA was evaluated in a retrospective observational study during an 18-month period, i.e. from March 2016 up to August 2017. All non-critically ill hospitalized patients and patients admitted at the day care hospital during the study period were included in this analysis.

#### Quantitative evaluation of preliminary results

A quantitative evaluation was performed by documenting the number of prescriptions that were checked by the hospital pharmacist (i.e. the number of clinical rule alerts) and the number of actions on alerts. Alerts were considered as clinically relevant whenever an action (electronic note with or without a phone call) was performed by the pharmacist. The analysis was also carried out without taking into account the automatic notes for potential sequential therapy.

The same evaluation was done for the following specific pharmacotherapeutic categories: drug use in renal insufficiency, drugs with high potential of QTc interval prolongation, drugs with a restricted indication or dosing and overruled very severe DDIs.

#### Qualitative evaluation of preliminary results

For the abovementioned four specific categories, patients’ mean age, the top five of the involved wards and the top five of the involved drugs were evaluated, for both the actions for which electronic notes vs. electronic notes plus phone calls were carried out. The age and wards were assessed in order to get an idea whether these patient populations are different from the ones for which we have bedside clinical pharmacy services.

#### Acceptance rate

An evaluation of the acceptance rate by the treating physician was performed. 300 unique patients for which an automatic note for sequential therapy was sent, 300 unique patients for which the pharmacist had put a note in the patient’s file for four other categories (drug use in renal insufficiency, drugs with high potential of QTc interval prolongation, drugs with a restricted indication or dosing and overruled very severe DDIs) and 300 unique patients for which the pharmacist had called the treating physician in combination with leaving an electronic note for the same four categories, were randomly selected during the 18-month period. Patients’ electronic medical records were explored retrospectively to evaluate whether the pharmacists’ advice had been followed. Acceptance was defined as a modification of a prescription (stop or dose correction) or a further follow-up of clinical and/or laboratory parameters in function of the specific flowchart within 72 h after the pharmacist’s action. Reasons for non-compliance with the pharmacist’s advice were not investigated. This evaluation was primarily carried out by one pharmacist (CQ). In case of doubt, a meeting was organized to reach consensus together with a second senior hospital pharmacist (IS) in order to take the final decision.

#### Statistical analysis

For this evaluation only descriptive analyses were performed.

## Results

### Quantitative evaluation of preliminary results

**Fig. 1:**
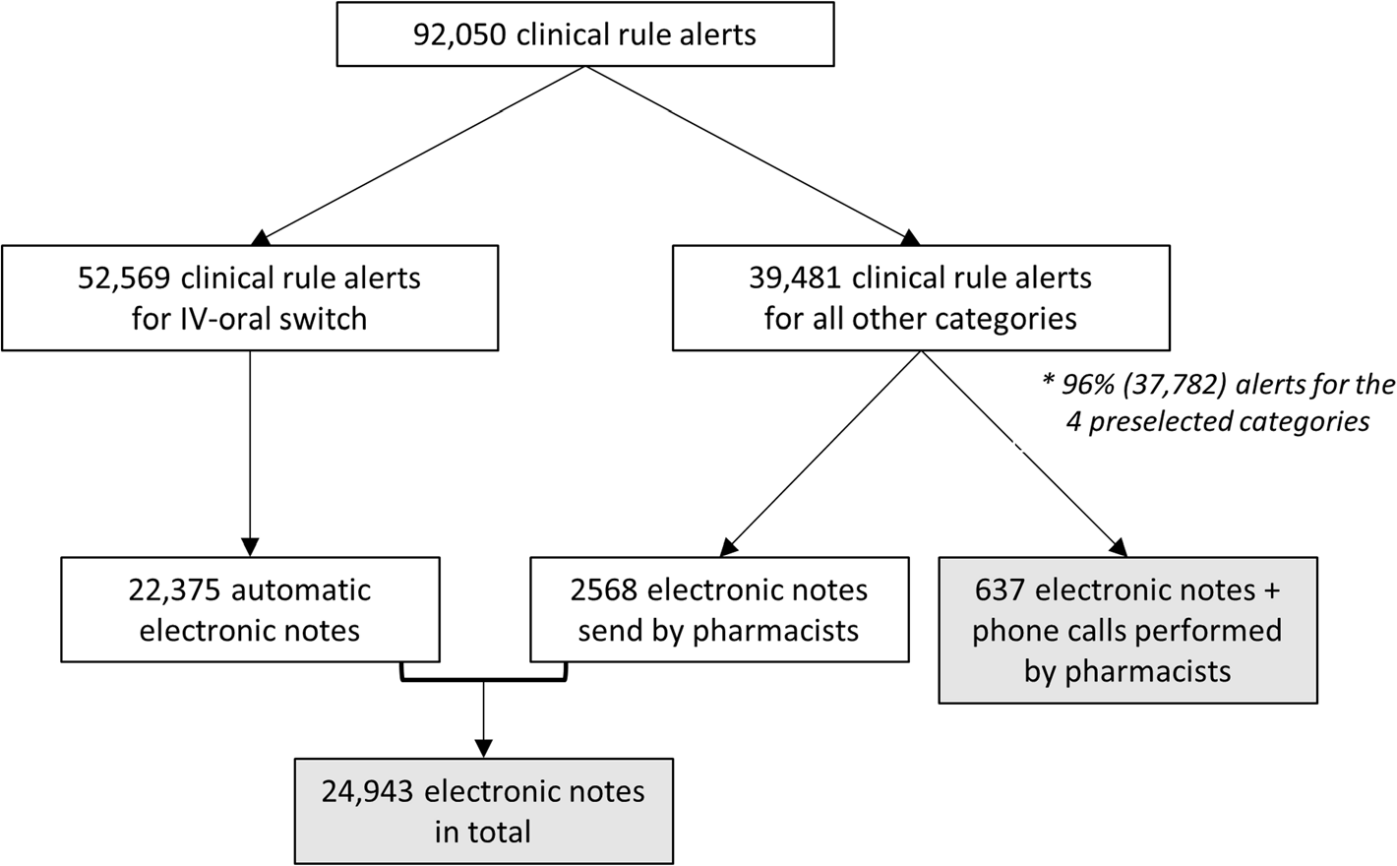
Flow diagram of the quantitative evaluation. *4 preselected categories: drug use in renal insufficiency, drugs with high potential of QTc interval prolongation, drugs with a restricted indication or dosing and overruled very severe DDIs

During the 18-month study period, 92,050 clinical rule alerts were extracted for which 24,943 (27%) electronic notes were sent and 637 (0.7%) electronic notes supplemented by phone calls were carried out. When analysed without the automatic warnings for sequential therapy, 39,481 clinical rule alerts were checked for which 2568 (7%) electronic notes were sent and 637 (1.6%) electronic notes plus phone calls were carried out (Fig. 1).

Table 2 shows the results for the four specific pharmacotherapeutic categories that were analysed in more detail. In total, 37,782 checks were carried out for these preselected categories, counting for 96% of the clinical rule alerts without taking into account the automatic notes for potential sequential therapy (Fig. 1; Table 2).

**Table 2:** Number of checks and actions during the 18-month study period for four pharmacotherapeutic categories^b^

Category	Number of prescriptions checked (n)	Number of electronic notes (n (%))	Number of electronic notes + phone calls (n (%))
Drug use in renal insufficiency	9381	444 (4.7%)	81 (0.9%)
Drugs with high potential of QTc prolongation	4223	608 (14%)	139 (3.3%)
Drugs with restricted indication or dosing	5276	448 (9%)	142 (2.7%)
Overruled very severe DDIs	18,902	939 (5%)	259 (1.4%)
*Total*	*37,782*	*2439 (7%)*	*621 (1.6%)*

^b^*DDI* drug-drug interaction, *n* absolute number

### Qualitative evaluation of preliminary results

Table 3 shows that for the four preselected categories the mean age of patients for which an electronic note was sent, was between 46.7 up to 74.2 years of age. Actions were frequently carried out for patients admitted on three wards, i.e. the emergency department, vascular and cardiac surgery. When looking into the drugs for which notes or phone calls were made, anticoagulants are frequently involved (cf. Table 3). In the evaluation of drugs used in renal insufficiency, 57% of electronic notes were sent for direct oral anticoagulants (DOACs) (10%) and low molecular weight heparins (LMWHs) (47%). Of the phone calls in this category, 73% were carried out for anticoagulants of which 21% was carried out for DOACs and 54% for LMWHs (total percentages not shown in Table 3). When focusing on the evaluation of overruled DDIs, the overruled combination of DOACs with other anticoagulants (often LMWHs) was the most frequent alert for which a phone call was carried out (37%) or an electronic note was sent (15%). The evaluation of the actions performed for the category of drugs with a restricted indication or dosing, revealed a high percentage of recommendations carried out for high dose meropenem (2 g, q8h).

**Table 3:** Detailed characteristics for four pharmacotherapeutic categories for which notes vs. notes plus phone calls were performed^c^

	Electronic note			Electronic note + phone call		
Category	Patient’s age in years (mean ± SD)	Top 5 patients’ wards (% of actions)	Top 5 drugs (% of actions)	Patient’s age in years (mean ± SD)	Top 5 patients’ wards (% of actions)	Top 5 drugs (% of actions)
Drug use in renal insufficiency	74 ± 15	1. ED (33%) 2. Nephrology (9%) 3. Vascular sg (8%) 4. Cardiac sg (6%) 5. Pneumology (5%)	1. Enoxaparin (43%) 2. Spironolactone (13%) 3. Metformin (13%) 4. Allopurinol (9%) 5. Vancomycin (5%)	72 ± 15	1. ED (38%) 2. Nephrology (9%) 3. Abdominal Tx (9%) 4. Vascular sg (9%) 5. Cardiac sg (9%)	1. Enoxaparin (48%) 2. Spironolactone (14%) 3. Rivaroxaban (14%) 4. Vancomycin (6%) 5. Tinzaparin (6%)
Drugs with high potential of QTc prolongation	70 ± 16	1. ED (19%) 2. Vascular sg (4%) 3. Hematology Tx (4%) 4. Cardiac sg (4%) 5. Hematology (4%)	1. Levofloxacin (36%) 2. Ondansetron (14%) 3. Fluconazol (12%) 4. Haloperidol (10%) 5. Sotalol (9%)	74 ± 15	1. ED (19%) 2. Cardiology (10%) 3. Geriatrics (6%) 4. Thoracic sg (6%) 5. Internal medicine (5%)	1. Levofloxacin (40%) 2. Fluconazol (12%) 3. Moxifloxacin (12%) 4. Ondansetron (9%) 5. Sotalol (9%)
Drugs with restricted indication or dosing	47 ± 27	1. Pediatric hematology-oncology (13%) 2. Pneumology (11%) 3. ED (9%) 4. Daycare ward pediatrics (4%) 5. Trauma sg (4%)	1. Meropenem high dose (36%) 2. Linezolid indication (17%) 3. Colistin dose (17%) 4. Temocillin indication (14%) 5. Rifampin indication (10%)	50 ± 24	1. Pneumology (16%) 2. ED (10%) 3. Pediatric hematology-oncology (8%) 4. Septic orthopedic sg (4%) 5. Trauma sg (4%)	1. Meropenem high dose (32%) 2. Colistin dose (20%) 3. Linezolid indication (14%) 4. Temocillin indication (14%) 5. Rifampin indication (9%)
Overruled very severe DDIs	70 ± 16	1. Thoracic sg (11%) 2. ED (8%) 3. Trauma sg (5%) 4. Urology (5%) 5. Vascular sg (4%)	1. QTc prolonging drugs^d^ & antiarrhythmic drugs (29%) 2. Factor Xa inhibitors & anticoagulants (15%) 3. Antiarrhythmic drugs & antipsychotics (12%) 4. Antiarrhythmic drugs & non-selective antidepressants (10%) 5. Metformin & contrast agents (5%)	72 ± 14	1. Trauma sg (10%) 2. Vascular sg (8%) 3. ED (8%) 4. Internal medicine (7%) 5. Urology (7%)	1. Factor Xa inhibitors & anticoagulants (37%) 2. QTc prolonging drugs^d^ & antiarrhythmic drugs (17%) 3. Antiarrhythmic drugs & antipsychotics (11%) 4. CYP3A4 substrates & CYP3A4 inhibitors (8%) 5. Antiarrhythmic drugs & non-selective antidepressants (5%)

^c^*SD* standard deviation, *DDI* drug-drug interaction, *ED* emergency department, *sg* surgery, *Tx* transplantation, *CYP3A4* cytochrome P450 3A4.

^d^QTc prolonging drugs: e.g. selective serotonin reuptake inhibitors, anticancer agents (e.g. sodium arsenite, anagrelide, eribulin), anti-emetics (e.g. 5-HT3-antagonists, domperidone), fluconazole, hydroxyzine, indapamide, ivabradine, vardenafil, bedaquilline

### Acceptance rate

The sample of 300 unique patients for which an electronic alert was sent for sequential therapy resulted finally in 341 actions, as in some patients there was more than one bio-equivalent drug. Of these 341 actions, 112 (33%) led to a switch from IV to oral therapy. For 64 (19%) of the electronic notes, the IV drug was stopped and for 165 (48%) of the notes, the advice was not accepted.

The sample of 300 unique patients for which the pharmacist left an electronic note in the patient’s file for the four preselected categories, resulted finally in 229 actions after excluding duplicates and notes for which the acceptance rate could not be verified because of a discharge or transfer to another hospital unit. Among the 229 pharmacy notes, 129 (56%) were accepted by the physician.

The sample of 300 unique patients for which the pharmacist had called the treating physician in combination with leaving an electronic note in the patient’s file for the same four categories, resulted also in 229 actions after the same exclusion. Among the 229 pharmacy phone calls, 189 (83%) were accepted by the physician.

Tables 4 and 5 shows the acceptance rate of notes and notes supplemented by phone calls for each category. In total, 318 (69%) out of 458 actions led to a documented modification in therapy or follow-up of clinical and/or laboratory parameters.

**Table 4:** Acceptance rate of actions for four pharmacotherapeutic categories performed by electronic notes^e^

Category	Total number of patients (n)	Exclusion		Total number of actions (n)	Number of actions accepted by physician (n (%))
		Duplicates	Not possible to verify*		
Drug use in renal insufficiency	61	10	6	45	31 (69%)
Drugs with high potential of QTc prolongation	78	4	11	63	34 (54%)
Drugs with restricted indication or dosing	51	3	5	43	21 (49%)
Overruled very severe DDIs	110	25	7	78	43 (55%)
*Total*	*300*	*71*		*229*	*129 (56%)*

^e^*DDI* drug-drug interaction, *n* absolute number. *notes for which the acceptance rate could not be verified because of a discharge or transfer to another hospital unit

**Table 5:** Acceptance rate of actions for four pharmacotherapeutic categories performed by electronic notes plus phone calls^f^

Category	Total number of patients (n)	Exclusion		Total number of actions (n)	Number of actions accepted by physician (n (%))
		Duplicates	Not possible to verify*		
Drug use in renal insufficiency	27	/	5	22	16 (73%)
Drugs with high potential of QTc prolongation	66	/	3	63	48 (76%)
Drugs with restricted indication or dosing	66	/	6	60	44 (73%)
Overruled very severe DDIs	141	46	11	84	81 (96%)
*Total*	*300*	*71*		*229*	*189 (83%)*

^f^*DDI* drug-drug interaction, *n* absolute number. *notes for which the acceptance rate could not be verified because of a discharge or transfer to another hospital unit

## Discussion

During the study period, a back-office CMA service, embedding 0.5 FTE hospital pharmacists, yielded 24,943 electronic notes and 637 electronic notes supplemented by phone calls, concerning potentially very harmful ADEs or DRPs. When analysed without the automatic warnings for sequential therapy, 96% of the checks were performed in four specific pharmacotherapeutic categories: drug use in renal insufficiency, drugs with high potential of QTc interval prolongation, drugs with a restricted indication or dosing and overruled very severe DDIs. Acceptance rate of pharmacy notes and pharmacy notes supplemented by phone calls, carried out in the abovementioned categories, was 56% and 83%, respectively.

As expected, geriatric patients (> 75 years) and children (< 18 years) were only rarely included, since bedside clinical pharmacy services are already provided on a daily base at both wards. Patients admitted at emergency and cardiac surgery departments were most frequently involved. These results contribute to a risk based assessment in order to prioritize future investments in bedside clinical pharmacy services at these specific wards. Anticoagulants, mentioned in 6 of the 78 clinical rules, are a major drug class for which advices were formulated. These results suggest that current knowledge on the potential dangers of (the novel oral) anticoagulants, when used in patients with decreased renal function or when used in combination with other anticoagulants, is lacking. These results indicate that it is worthwhile to look for potential ADEs with these agents. Concerning other issues revealed by CMA, action was already undertaken e.g. for the use of meropenem high dose (2 g, q8h) a newsletter was sent to the pediatric hematology-oncology ward.

Our results indicate that the implementation of CMA is a significant addition to the standard services provided by the currently implemented basic CDSS, which is running on a hospital wide basis but only supporting at the step of prescribing without taking into account relevant laboratory values, and also to bedside clinical pharmacy services, which provides support at any time of the treatment but only runs for a limited and highly selected patient population. CMA, which combines automated screening of multiple data sources in the patient’s electronic medical record with the back-office evaluation of the patient’s therapy by a trained hospital pharmacist, has the ability to provide pharmacotherapeutic support on a hospital wide basis. With maximum integration of patient specific characteristics with details on drug treatment, a personalized advice can be given. The organization of our CMA service was well considered before implementation and is based on algorithms, which rely on evidence-based literature and practice-based experience, user-friendly flowcharts and uniform advices which need approval by the P&T committee before implementation. The obtained kappa value (0.79) also proves that there is a homogeneity between the pharmacists in checking the high risk prescriptions.

There are a lot of studies describing the implementation and evaluation of CPOE, basic and advanced CPOE/CDSS, but there are few studies [10, 23–26] describing services like the CMA, consisting of alerts aiming exclusively at pharmacists, followed by pharmacists’ performed actions in case of medication inappropriateness. Our CMA system is very similar to the pharmacy adverse drug event alerting system (ADEAS) developed by Rommers et al. [10] They formulated a comparable list of clinical rules (*n* = 121) divided in risk categories. Like CMA, the generated alerts go to the pharmacist and not directly to the prescribing physician to prevent alert fatigue with the physician. They use ADEAS as a tool for the hospital pharmacist for more clinical ward-based activities, whilst CMA is developed as a back-office clinical service [10]. In our opinion, a pharmacy decision support system like CMA and ADEAS is an essential added value to support medication surveillance and pharmacotherapy in European countries in which healthcare budget supporting bedside clinical pharmacy is scarce. Since clinical rules generally rely on literature-based evidence and practice-based evidence, we believe that the implementation of a similar service is possible in other European centers.

Our current adoption of the CMA service still demonstrates some limitations. First, when looking into the results without the automated suggestions for sequential therapy, the ratio of actions (*n* = 3205) to the total number of clinical rule alerts (*n* = 39,481) is low. Only 8% of the alerts were considered clinically relevant by the hospital pharmacists, which implies a large number of false positive alerts and unnecessary checks by the hospital pharmacist. This number of irrelevant alerts seems high, but a comparable percentage has also been observed in some previous studies which investigated one or more specific advanced medication alerts [12, 13, 23–25]. In the study of Rommers et al., a similar percentage (7.8%) of the alerts resulted in advice to prevent possible ADEs [23]. The reasons why clinical rule alerts were classified as not relevant by the hospital pharmacist were not systematically analysed in our study. However some reasons for non-relevance were frequently identified: 1) the dosage was already adjusted, 2) the drug was (temporarily) stopped, 3) the monitored laboratory value or clinical parameter was already reverted to within the reference limits, 4) repeated alerts which were already evaluated by the pharmacist and 5) double alerts with the same content. The same reasons were already mentioned by de Wit et al. and Rommers et al. [12, 23] In the near future we plan to increase specificity of the CMA by further reformulating and fine-tuning the alert criteria of the clinical rules (e.g. by identifying and integrating more patient characteristics or parameters like weight, specific dose regimens, clinical symptoms etc.). Unfortunately, the inclusion of more alert criteria is limited because some patient characteristics are not structurally electronically documented in the medical record. So further automation is dependent on the digitalization of patients’ characteristics, which was already mentioned by Eppenga et al. [13] To prevent repeated alerts, the alert could be suppressed until a relevant change of one of the alert criteria makes the alert reappear. Already reverted laboratory values could be a result of delay in data delivery, which can be resolved by integration of the CMA in HIS resulting in a direct link with the laboratory system. Furthermore, we also have to reconsider the basic CDSS for DDIs. The limited number of actions carried out for overruled DDIs indicates that many of these DDIs have to be overruled by the physician without a high risk for the patient. These unnecessary overrules might definitely lead to frustration and alert fatigue for the prescribing clinicians. For these interactions, it should be considered to omit the overrule by decreasing the severity score and/or by reassessing and reformulating the time interval of the defined interaction in CDSS.

Second, the acceptance rate of the automatically electronic notes suggesting a switch from an IV to oral formulation of a bio-equivalent drug is relatively low: only 33% of the automated notes led to a therapy switch. Therefore, the content of the alert criteria and the validity of the automatic screening algorithm for sequential therapy should be evaluated.

Additionally, for the four selected domains, a total acceptance rate of 69% was observed. This in in line with the ADEAS study, where 128 (63%) out of 204 actions led to a documented modification in therapy [23].

However, for the alerts for which only an electronic notes was sent for the four selected domains, a moderate acceptance level of 56% was seen. This implies that almost half of the advices was not accepted or perhaps not read by the physician. Along with the reasons mentioned by clinicians to deny the advice, the clinical relevance of clinical rules for which an advice is usually not accepted needs to be assessed. In the near future, a satisfaction survey is planned for physicians to evaluate their general experiences with this service, their overall reasons for agreeing or disagreeing with the pharmacotherapeutic advice and their specific wishes or comments for future expansion.

In contrast, the majority of advices given by a phone call on top of the electronic note was accepted (83%). This was expected since a phone call is only performed for a medication order with a very high risk of ADE or DRP. It also indicates that an additional phone call may have a greater impact on the physician with a higher acceptance rate. Therefore it should be considered whether a phone call should be carried out faster.

Third, the acceptance level may also have been overestimated which is inherent to the retrospective evaluation of the acceptance rate: in some cases the physician could have modified the prescription independently of the pharmacy alert.

In conclusion, a number of benefits but also limitations were identified aiming for the optimization of CMA. Next to further improving the CMA (focusing on increasing the specificity and reconsidering the basic CDSS for DDIs), an expansion of the service is planned to cover a much wider range of drugs or drug classes and to cover more patient groups with the aim of avoiding even more preventable ADEs. First, clinical rules focusing on a specific pharmacotherapeutic domain will be developed, i.e. focusing on antimicrobial stewardship, anticoagulation therapy, post-operative pain management and total parenteral nutrition therapy. Second, as it has been described in literature that critically ill patients are more prone to suffer from ADEs, an ICU-focused implementation of the CMA service is also planned. Lastly, future research (i.e. an interrupted time series analysis) is foreseen to measure the additional effect of the advanced clinical rules on top of basic CPOE/CDSS and bedside clinical pharmacy in preventing PIMs in a controlled design. After this analysis, we need to reconsider if some alerts might be better situated as CDSS warnings to support physicians at the moment of prescribing in case immediately action is necessary.

## Conclusion

CMA involves trained hospital pharmacists who daily check high risk prescriptions generated by advanced clinical rules integrating patient specific characteristics with information on medication. This service is valuable as it yielded a high number of pharmacists’ actions for clinically relevant alerts with an adequate acceptance rate by physicians. In our opinion, CMA could support medication surveillance and optimize pharmacotherapy in synergy with already integrated basic CPOE/CDSS and bedside clinical pharmacy. Otherwise, the study also highlighted a number of limitations, allowing further research and improvement of the service.

## Additional file

Additional file 1: Definite set of 78 advanced clinical rules. (DOCX 16 kb)

## Abbreviations

ADE: Adverse drug event
CDSS: Clinical Decision Support System
CMA: Check of Medication Appropriateness
CPOE: Computerized Physician Order Entry
DDI: Drug-drug interaction
DOAC: Direct oral anticoagulant
DRP: Drug-related problem
FTE: Full-time equivalent
HIS: Hospital Information System
ICU: Intensive care unit
IV: Intravenous
LMWH: Low molecular weight heparin
P&T: Pharmacy & Therapeutics
PIM: Potentially inappropriate medication
