# Supplementary material for: Development and implementation of “Check of Medication Appropriateness” (CMA): advanced pharmacotherapy-related clinical rules to support medication surveillance
Source: BMC Med Inform Decis Mak. 2019 Feb 11;19:29. doi: 10.1186/s12911-019-0748-5 (PMC6371500; doi:10.1186/s12911-019-0748-5)
Supplement: Supplementary file 1 — Definite set of 78 advanced clinical rules. (DOCX 16 kb) [file 12911_2019_748_MOESM1_ESM.docx]

Additional file 1. **Definite set of 78 advanced clinical rules**

| **Category** | **Clinical rule** |
| --- | --- |
| 1. | **Overrules of alerts for very severe DDIs generated by the CDSS** |
|  | Reduced effect of contraceptives by acetretin |
|  | Reduced effect of atovaquone by rifampin |
|  | Patient treated with combined use of allergen extract and ACE-inhibitor and/or β-mimetic agent leading to an increased risk of anaphylaxis |
|  | Patient treated with combined use of disulfiram and ethanol |
|  | Increased effect of ivabradine by CYP enzyme inhibitor leading to an increased risk of bradycardia |
|  | Patient treated with combined use of triptan and ergopeptide leading to an increased risk of excessive vasoconstriction |
|  | Patient treated with combined use of cardiac glycoside (digitalis) and Ca parenteral leading to an increased risk of digitalis intoxication |
|  | Patient treated with MAO inhibitor and an increased risk of hypertensive crisis |
|  | Patient treated with phosphodiesterase-5 enzyme inhibitor and an increased risk of severe hypotension |
|  | Increased effect of repaglinide by gemfibrozil leading to an increased risk of severe hypoglycemia |
|  | Increased effect of bupropion by MAO-inhibitors |
|  | Patient treated with combined use of ceftriaxone and Ca gluconate parenteral |
|  | Increased effect of statin by CYP enzyme inhibitor leading to an increased risk of rhabdomyolysis |
|  | Patient treated with combined use of different anticoagulant drugs (LMWH, VKA, DOAC) leading to an increased risk of bleeding |
|  | Patient treated with combined use of serotonergic drugs (SSRI, SNRI, MAO-I, TCA, triptans) leading to an increased risk of serotonin syndrome |
|  | Patient treated with a benzodiazepine (midazolam, triazolam) and an increased risk of respiratory distress |
|  | Increased effect of quetiapine by CYP enzyme inhibitor |
|  | Patient treated with combined use of Saccharomyces cerevisiae and immunosuppressive drug or corticosteroid leading to an increased risk of invasive fungal infection |
|  | Patient treated with combined use of different drugs that can cause neutropenia |
|  | Patient treated with combined use of different drugs that can cause QTc prolongation |
|  | Increased effect of bosentan by ciclosporin |
|  | Increased effect of eletriptan by CYP enzyme inhibitor |
|  | Increased effect of fluoropyrimidine by sorivudine or brivudine |
|  | Increased effect of quindine by azole derivative leading to an increased risk of torsades de points |
|  | Increased effect of azathioprine or mercaptopurine by xanthine oxidase inhibitor |
|  | Increased effect of tizanidine by CYP1A2 enzyme inhibitor |
|  | Patient treated with combined use of retinoid and tetracycline leading to an increased risk of intracranial hypertension |
|  | Vaccination with live attenuated vaccine in patient taken immunosuppressive drug |
|  | Reduced effect of valproic acid by carbapenems leading to an increased risk of convulsions |
| **2.** | **Drugs with a restricted indication or dosing** |
|  | Patient treated with abiraterone without daily corticosteroids |
|  | Patient treated with an amino acid/dipeptide (dipeptide alanyl-glutamine) infusion solution |
|  | Patient treated with idealisisb without PCP prophylaxis |
|  | Patient treated with high dose meropenem |
|  | Patient treated with temocillin |
|  | Patient treated with daptomycin |
|  | Patient treated with colistin given oral without gut decontamination |
|  | Patient treated with colistin given IV |
|  | Patient treated with linezolid |
|  | Patient treated with rifampin |
|  | Patient treated with tigecycline |
|  | Patient treated with chloramphenicol |
|  | Patient treated with thiamfenicol |
|  | Patient treated with vancomycin lock therapy without systemic antimicrobial therapy |
|  | Patient treated with ceftazidime lock therapy without systemic antimicrobial therapy |
|  | Patient treated with fidaxomicin |
| **3** | **Medication use potentially leading to biochemical changes** |
|  | Patient with a CrCl < 50 ml/min and treated with an NSAID |
|  | Patient with a CrCl < 30 ml/min and treated with atenolol |
|  | Patient with a CrCl < 30 ml/min and treated with metformin |
|  | Patient with a CrCl < 10 ml/min and treated with allopurinol (> 100 mg/day) |
|  | Patient with a CrCl < 30 ml/min and treated with enoxaparin |
|  | Patient with a CrCl < 30 ml/min and treated with apixaban |
|  | Patient with a CrCl < 50 ml/min and treated with rivaroxaban |
|  | Patient with a CrCl < 50 ml/min and treated with dabigatran |
|  | Patient with a CrCl < 30 ml/min and treated with spironolactone |
|  | Patient with a CrCl < 30 ml/min and treated with a thiazide diuretic |
|  | Patient treated with an aminoglycoside and a CrCl < 30 ml/min and an unknown trough level |
|  | Patient treated with vancomycin and a CrCl < 30 ml/min and an unknown trough level |
|  | Patient with an absolute neutrophil count < 1.5*10^9^/L and treated with clozapine |
|  | Patient with an absolute neutrophil count < 1.5*10^9^/L and treated with diaminodiphenyl sulfone |
|  | Patient with a K < 3.5 mmol/L and treated with a loop and/or thiazide diuretic without K-supplementation |
|  | Patient with a K < 3.5 mmol/L and treated with piperacillin (tazobactam) without K-supplementation |
|  | Patient with a K < 3.5 mmol/L and treated with flucloxacillin without K-supplementation |
|  | Patient with a K < 3.5 mmol/L and treated with liposomal amphotericin B without K-supplementation |
|  | Patient with a K > 5.5 mmol/L and treated with total parenteral nutrition |
|  | Patient with a K > 5.5 mmol/L and treated with K-supplementation (K chloride, K phosphate, K gluconate) |
|  | Patient with a K > 5.5 mmol/L and treated with an ACE inhibitor |
|  | Patient with a K > 5.5 mmol/L and treated with cotrimoxazole |
|  | Patient with a K > 5.5 mmol/L and treated with a K-sparing diuretic (spironolactone, canrenoate, eplerenone) |
|  | Patient with a K > 5.5 mmol/L and treated with triamterene |
|  | Patient with a K > 5.5 mmol/L and treated with amiloride |
|  | Patient with a supratherapeutic INR (INR > 4) and treated with total parenteral nutrition |
|  | Patient with a supratherapeutic INR (INR > 4) and treated with a VKA |
|  | Patient with a QTc > 450/470ms and treated with haloperidol |
| **4** | **Potential sequential therapy (IV to oral switch) for bio-equivalent drugs** |
| **5** | **Others** |
|  | Patient treated with non-crushable drugs administered through enteral feeding tube |
|  | Patient treated with total parenteral nutrition without weekly vitamin K |
|  | Patient treated with drug administered subcutaneous |

**^*^**DDI = drug-drug interaction, CDSS = clinical decision support system, ACE = angiotensin converting enzyme, CYP = cytochrome P450, MAO = monoamine oxidase, LMWH = low molecular weight heparin, VKA = vitamin K antagonist, DOAC = direct oral anticoagulant, SSRI = selective serotonin reuptake inhibitor, SNRI = selective noradrenaline reuptake inhibitor, TCA = tricyclic antidepressant, PCP = *Pneumocystis jirovecii* pneumonia, IV = intravenous, CrCl = creatinine clearance, NSAID = nonsteroidal anti-inflammatory drug, INR = international normalized ratio.
